# Exploring the collaborative care of patients with pelvic fractures in Tshwane, South Africa

**DOI:** 10.4102/safp.v65i1.5705

**Published:** 2023-05-26

**Authors:** Ntombenkosi A. Sobantu, Muziwakhe D. Tshabalala, Verusia Chetty

**Affiliations:** 1 Department of Physiotherapy, School of Health Care Sciences, Sefako Makgatho Health Sciences University, Pretoria, South Africa; 2 Department of Physiotherapy, Faculty of Health Sciences, University of KwaZulu-Natal, Durban, South Africa

**Keywords:** pelvic fractures, health-related quality of life, rehabilitation, healthcare, interprofessional collaboration, South Africa

## Abstract

**Background:**

Pelvic fractures are complex injuries that lead to long-term disabilities and poor health-related quality of life (HRQoL). Even though pelvic fractures are known to be challenging to manage, there is limited information on guidelines and protocols to ensure that patients receive comprehensive and collaborative healthcare.

**Methods:**

A qualitative descriptive phenomenological approach was utilised to explore current practices and innovations of healthcare professionals (HPs) in Tshwane academic hospitals in the collaborative management and rehabilitation of patients with pelvic fractures, using semi-structured interviews. Thematic analysis was used to analyse data.

**Results:**

Six overarching themes were identified from the interviews with HPs: The biopsychosocial lens of the patient, limitations in approaches to care, contextual impediments to care, the team challenge; the biopsychosocial aspects of care and forging forward to improve care.

**Conclusion:**

A multidisciplinary approach is encouraged for the comprehensive management of pelvic fractures. However, a poor understanding of roles and poor referral structures challenge this approach. Further barriers to caring include staff shortages and limited resources. Healthcare professionals recommended interprofessional education and collaborative practice, student training and using standardised outcome measurement tools to improve care for patients with pelvic fractures.

**Contribution:**

This study lays a foundation to initiate conversations about the development of an interprofessional model of care for patients with pelvic fractures. Findings might inform health policies on the management of pelvic fractures. Healthcare professionals might apply strategies that enhance the quality of healthcare provided. Patients with pelvic fractures might receive quality interprofessional healthcare that promotes quality of life, post pelvic fractures.

## Introduction

Pelvic fractures are less common injuries. However, their incidence is increasing because of the increase in the number of high-velocity impacts caused by motor vehicle accidents (MVAs) in low and middle-income countries.^1^ The trend also applies in South Africa (SA) (recently classified as an upper middle-income country). Palmcrantz et al.^2^ highlighted that the most common cause of pelvic fractures is MVA. Pelvic fractures are complex injuries because they seldom occur in isolation or as single fractures, but mostly as multiple injuries. Globally, the incidence of pelvic fractures ranges from 3% to 8% of all fractures.^3^ However, in SA the incidence of pelvic fractures is reported to be 16% of all major trauma injuries.^2^ Sobantu et al.^4^ in Tshwane, SA, reported that 80.5% of patients with pelvic fractures sustained poly-trauma, of which 29.7% had multiple pelvic fractures. Furthermore, only 19.5% sustained a single pelvic fracture.^4^ Studies in KwaZulu-Natal, SA,^5^ and in Bangladesh^6^ found similar results, which indicated that pelvic fractures were part of poly-trauma in the majority of cases. Pelvic fractures are usually concomitant with urological and musculoskeletal injuries.^7^

The complexity of pelvic fractures poses a challenge in managing patients with such injuries. These complexities may often present with residual impairments, which lead to disability post-acute management of pelvic fractures.^1^ They also lead to a poor health-related quality of life (HRQoL) in individuals.^1^ Many patients who have sustained pelvic fractures present with both physical and psychosocial impairments, making them less likely to live independent, economically productive lives.^8,9^ The consequences of pelvic fractures are especially dire in young adults because they end up being unable to earn an independent living or have a fulfilling family life. This is the case whether patients have sustained a single or multiple pelvic fractures. Following discharge, patients with pelvic fractures have reported that they were struggling to perform day-to-day activities, which included household chores, being involved in the community and earning an income because of chronic pain and physical and emotional challenges.^4^ Furthermore, both physical and mental health are substantially affected in patients who have sustained pelvic fractures, leading to a significant burden on the country’s economy.^8,10^ It can, therefore, be concluded that pelvic fractures are injuries that contribute to the burden of disease in any country and are one of the causes of years lived with disability (YLDs).^11^

The management of pelvic fractures should adopt a biopsychosocial model of healthcare and early multidisciplinary intervention that includes a comprehensive rehabilitation programme to improve recovery in patients with pelvic fractures.^3^ Patients with pelvic fractures will likely recover well and achieve better health outcomes with early, integrated interventions.^12^ Furthermore, interprofessional collaboration and community involvement should be encouraged^13^ to ensure that intervention for patients with pelvic fractures continues beyond the acute phase. Interdisciplinary healthcare, which involves psychological intervention early in the acute phase, is advocated for patients with pelvic fractures until they recover.^8^ However, there is limited literature on guidelines and protocols for managing and rehabilitating patients who have sustained pelvic fractures. Hence, this study aims to explore the current practices and innovations of healthcare professionals (HPs) in Tshwane academic hospitals in the collaborative management and rehabilitation of patients who sustain pelvic fractures. Findings from this study will contribute to the development of an interprofessional model of care for patients with pelvic fractures in academic hospitals within Tshwane, SA.

## Methods

### Study design

A qualitative descriptive phenomenological approach was utilised to explore the current practices and innovations of HPs in Tshwane academic hospitals in the collaborative management and rehabilitation of patients with pelvic fractures. Semi-structured interviews were conducted with the HPs. A phenomenological approach was chosen to explore the lived, individual experiences of HPs.^14^ The phenomenological construct promotes understanding and explanation of the social world through the lived experiences of others and allows stakeholders such as HPs to learn from each other.^14^ The interviews remained flexible and adaptable, facilitated in-depth conversations, and allowed follow-up on responses that were superficial or unclear, providing new, relevant information.

### Participants and materials

Participants were purposively sampled HPs from three Tshwane academic hospitals in SA who were managing patients with pelvic fractures. A maximum variation sampling procedure was used to ensure that the selected professionals gave detailed, rich information relating to their experiences in the management and rehabilitation of patients with pelvic fractures. A maximum variation sampling procedure allowed involvement of professionals with different perspectives in managing patients with pelvic fractures.

Recruitment was performed through the designated healthcare facility authorities, who recommended and invited health professionals who manage patients with pelvic fractures. The recommended professionals were contacted by telephone and/or email and provided with details of the study, and suitable times for interviews were arranged. Healthcare professionals were recruited from different departments. The HPs were both male and female from varied ethnic backgrounds and included nurses, orthopaedic doctors, urologists, physiotherapists, dieticians, social workers, trauma surgeons, occupational therapists, orthotists and prosthetists, gynaecologists and psychologists.

To ensure the rigour of the interview guide, the interview guide was developed by the authors guided by the literature and then amended following a pilot, one-on-one, in-depth, semi-structured interview with an academic who had vast experience in the rehabilitation of patients with orthopaedic and sports injuries. The interview questions focused on describing the current approaches, strategies and challenges and suggested innovations in the collaborative management and rehabilitation of patients with pelvic fractures. The interviewer was an academic HP who is conversant in conducting semi-structured interviews because of previous involvement in qualitative research studies.

Sixteen semi-structured interviews were conducted, and data collection ceased when no new data emerged from participants. Interviews lasted between 44 min and 78 min and were video-recorded following consent from participants. The long engagement with each participant was to improve trust and rapport between the researcher and the participant and ensure that all aspects of the topic were explored. Long engagement also allowed participants to volunteer even more information, which was crucial for the study. Participants were relaxed in their own environment and seemed eager and willing to share information. The interviews were conducted between June 2021 and March 2022. Interviews remained flexible and participants were allowed to explore various themes with the interviewer.

### Data handling and analysis

The recordings were transcribed verbatim immediately afterward by a research assistant with experience in transcription. Trustworthiness strategies utilised in this study included: member checking, long engagement, thick description and fidelity to data analysis with co-coding of data by the authors. Seven transcripts were sent to the respective participants for member checking to ensure that discussions were captured accurately. Findings from the transcripts were thematically analysed.^15^ This method followed six steps: familiarising one with the data, generating initial codes, searching for themes, reviewing potential themes, defining and naming themes, and producing the report.^15^ The authors familiarised themselves with the data; initial codes were generated and data were reviewed for potential themes. The long engagement allowed for a thick description during data analysis and interpretation. Finally, the authors agreed on the definition and naming of the overarching themes. The findings are discussed under six themes.

### Ethical considerations

Ethical clearance for the research study was obtained from the University of KwaZulu-Natal (UKZN) (HSSREC/00001434/2020) and the University of Pretoria (UP) (606/2020); the National Department of Health (NDoH) (NDoH_202008_011); the provincial Department of Health (PDoH) (GP_202012_032) and hospital chief executive officers at the research sites, before the study commenced. All participants signed an informed consent before the interviews.

## Results

### Participant demographic characteristics (*N* = 16)

Four physiotherapists, two occupational therapists, one dietitian, one trauma surgeon, four orthopaedic surgeons, one orthoptist or prosthetist, one social worker, one professional nurse and one urologist participated in the study. Table 1 reflects the details of the HPs.

**TABLE 1:** Participants’ characteristics (*N* = 16).

Participants	Gender	Healthcare professionals	Clinical experience (years)
Participant 1	Female	Physiotherapist	36
Participant 2	Female	Physiotherapist	14
Participant 3	Female	Occupational therapist	02
Participant 4	Female	Physiotherapist	06
Participant 5	Male	Occupational therapist	15
Participant 6	Female	Physiotherapist	20
Participant 7	Female	Dietitian	14
Participant 8	Male	Trauma surgeon	30
Participant 9	Male	Orthopaedic surgeon	05
Participant 10	Female	Orthopaedic surgeon	08
Participant 11	Male	Orthopaedic surgeon	17
Participant 12	Female	Orthotist or prosthetist	11
Participant 13	Male	Social worker	10
Participant 14	Male	Orthopaedic surgeon	07
Participant 15	Female	Professional nurse	28
Participant 16	Male	Urologist	30

These participants (Table 1) shared their perspectives on their lived experiences of the approaches, strategies and challenges, and envisaged innovations in the management and rehabilitation of patients with pelvic fractures. Six main themes were identified from their interviews: the biopsychosocial lens of the patient, care approach limitations, contextual impediments to care, the team challenge, the biopsychosocial aspects of care and forging forward to improve care. The six themes and their sub-themes are presented in Table 2, which gives a clear guide on how the results are presented.

**TABLE 2:** Findings on collaborative care for patients with pelvic fractures.

Themes	Sub-themes
1. The biopsychosocial lens of the patient	1.1 Impairments experienced by the patient 1.2 Inhibitors to quality of life 1.3 Social barriers 1.4 Psychological influence
2. Care approach limitations	2.1 The lack of comprehensive evaluations and guidelines for assessment and treatment 2.2 Poor use of standardised outcome measurement tools
3. Contextual impediments to care	3.1 Staff shortages impacting the quality of care 3.2 Shortage of beds and early discharge of patients 3.3 Limited resources and financial barriers
4. The team challenge	4.1 The lack of understanding of different health professional roles 4.2 Repercussions of late management and poor referral structure 4.3 The lack of collaborative teamwork 4.4 Continuity of care after discharge 4.5 Joint appointment of health professionals (hospital and academia)
5. The biopsychosocial aspects of healthcare	5.1 Physical rehabilitation 5.2 Medication 5.3 Psychological approach to care 5.4 Social support
6. Forging forward to improve care	6.1 Interprofessional education and collaborative practice 6.2 Student training 6.3 Adopting the International Classification of Function 6.4 Early management 6.5 Patient-centred continued care 6.6 Patient education 6.7 Normalisation of use of outcome measurement tools 6.8 Awareness campaigns

### Themes and subthemes

Six themes emerged from the interviews. Table 2 presents the six themes and highlights their respective sub-themes.

### Theme 1: The biopsychosocial lens of the patient

This theme highlights the HPs’ perception of impairments and their influence on the function and quality of life (QoL) of patients with pelvic fractures. It included impairments experienced by the patient, inhibitors to QoL, social barriers and psychological influence as sub-themes.

Participants highlighted impairments experienced by the patients they cared for:

‘I get referred patients that, after getting pelvic fractures, they usually experience pelvic floor dysfunction. In males, usually, you find that they are struggling with urinary incontinence and also erectile dysfunction. And then in women, it is usually pelvic pain with sexual intercourse and also incontinence.’ (Participant 2, female, physiotherapist)

‘… They [*patients*] tend to have a lot of chronic pelvic pain … and this pain can be so disabling that they resort to using some sort of substance.’ (Participant 14, male, orthopaedic surgeon)

Some participants identified inhibitors to QoL:

‘… we are discharging patients, but yet these patients are not independent … They are unable to actually function independently, or their score is poor.’ (Participant 5, male, OT)

‘Erectile dysfunction yes, related to pelvic fractures for sure. Ejaculatory issues probably related to that as well and the sexual feelings … They [*females*] can have problems with vaginismus pain, dyspareunia … It impacts massively on the quality of life if there’s sexual dysfunction, both for females and males …’(Participant 16, male, urologist)

Most participants highlighted social barriers that negatively influence the patients’ recovery:

‘… the family … the support system of the person … who sustained a pelvic fracture, it’s not supportive towards the person, or they are not aware of the severity of the diagnosis … of the injury … Sometimes they [*patients*] experience anger and they end up not cooperating … Because if you are injured, you will automatically grieve. Your role at home will change. Your emotional state will be disturbed; your psychological state will be disturbed.’ (Participant 13, male, social worker)

‘… some patients do not have family members or someone to look after them at home.’ (Participant 4, female, physiotherapist)

The participants identified psychological issues in the patients, post-injury:

‘… some get post-traumatic stress disorder, some are getting worried that they left a family at home, get all sorts of depressive episodes, some even to an extent of developing acute psychosis.’ (Participant 14, male, orthopaedic surgeon)

‘… They will feel sad. They will feel pained by their state. Sometimes they experience anger and they end up not cooperating. And lack of cooperation means it will affect the length of stay. Even the rehab, it will be affected. Because if you are injured, you will automatically grieve. Your emotional state will be disturbed; your psychological state will be disturbed.’ (Participant 13, male, social worker)

### Theme 2: Care approach limitations

The care approach limitations included two sub-themes: the lack of comprehensive evaluations and guidelines for assessment and treatment and poor use of standardised outcome measurement tools (OMTs).

Some participants indicated that both the subjective and objective assessments ended up being very limited, and they were unsure if there were guidelines to follow when managing patients with pelvic fractures. The following quote highlights this phenomenon:

‘Because the physio does not ask questions regarding incontinence and regarding pain with sexual intercourse, regarding any of that, usually they do not pick it up that the patient has such problems.’ (Participant 2, female, physiotherapist)

The majority of the participants emphasised poor use of standardised OMTs in their approach to care:

‘… It [*the goal*] is just to make sure and to strive to make the patient as independent as possible and to not be like a burden on their caregivers. So that would be our main measurement tool.’ (Participant 3, female, OT)

‘… We do not really have an outcome measure …’ (Participant 4, female, physiotherapist)

### Theme 3: Contextual impediments to care

Contextual impediments to care include staff shortages impacting the quality of care, the shortage of beds and early discharge of patients, and limited resources and financial barriers as sub-themes.

Participants highlighted that staff shortages impact the quality of care:

‘… The ones [*standardised tools*] that we have, we do not even get to use them because of the timelines that we have. We are constantly under pressure. You need to discharge. You do not have the luxury to actually do these standardised tests so that you can measure and have good or proper records of functional outcomes.’ (Participant 5, male, OT)

‘It is shortage of staff, and the other thing is, there are too many activities in the orthopaedics.’ (Participant 6, female, physiotherapist)

Participants identified the shortage of beds and the early discharge of patients:

‘… Most of the time, they [*doctors*] are concerned about the bed, so as soon as the patient is able to walk independently, we discharge the patient.’ (Participant 6, female, physiotherapist)

‘… You do get a few that do not achieve their outcomes by the time that we do discharge them because we are pressed for beds most of the time as well …’ (Participant 4, female, physiotherapist)

Participants highlighted limited resources and financial barriers that hinder healthcare:

‘I think it’s access to a specific equipment and specialist care when it comes to urology … There are some specific types of cystoscopies that we prefer … The point is that a lot of these equipment are slightly expensive.’ (Participant 16, male, urologist)

### Theme 4: The team challenges

The team challenges included the lack of understanding of different HP roles, a poor referral structure, the repercussions of late management, a lack of collaborative teamwork and continuity of care after discharge as sub-themes.

Some participants emphasised a lack of understanding of different HP roles:

‘They [*doctors*] think pelvic fractures – and they just confine it to physio. Which is not the case …’ (Participant 5, male, OT)

‘Because back then people would have that perspective that “No orthotist, they will be our last resort”.’ Participant 12, female, orthotist)

A lack of collaborative teamwork among HPs was a common phenomenon. Many participants believed that professionals worked in silos during the management and rehabilitation of patients with pelvic fractures:

‘… Some units do not involve urology unless the patient has symptoms. The danger of that is that you may end up with incontinence in children and in females. You can get the extraperitoneal rupture being missed, which may cause a sepsis in the patients.’ (Participant 16, male, urologist)

‘And then the psychologist, as well, because lying in bed for eight or six weeks, I think it can be depressing at some point, so involving a psychologist, it will also help as well. The social workers … only come when there is a need, like maybe if there are social issues at home.’ (Participant 6, female, physiotherapist)

Many participants emphasised the repercussions of late management and poor referral structures that lead to poor patient outcomes:

‘If we do not get them on time, they present with complications. So, I think that is where the challenge is …’ (Participant 6, female, physiotherapist)

‘… Once we delay …, it becomes very difficult to operate those patients, and … the outcomes are worse compared to the ones we operated earlier …’ (Participant 9, male, orthopaedic surgeon)

Some participants were concerned that patients are discharged home even when they still need further rehabilitation post-acute phase:

‘… the physio is done while the patient is in hospital, but ideally that patient should be sent to a rehabilitation centre, once the patient is ready to be discharged from hospital. But we do not have that facility in the state-run services so, what we see and our experience, … is that the patient still requires active rehabilitation and he cannot do it. You need to send him home even with a wheelchair or with crutches because there is no rehabilitation hospital.’ (Participant 8, male, trauma surgeon)

### Theme 5: The biopsychosocial aspects of healthcare

The biopsychosocial aspects of healthcare included physical rehabilitation, psychological approaches to care and social support as sub-themes.

Participants highlighted physical rehabilitation as one aspect of healthcare:

‘It [*goal*] is mainly remediating, where we improve the affected body functions – your range of motion, your muscle strength, endurance … And once the patient is able to stand, able to walk, do the basic ADL, which is, they are able to stand, walk to the hand basin to brush their teeth, they are able to wash their face, eat, climb the stairs, in and out of the bathtub …’ (Participant 5, Male, OT)

Most participants highlighted the importance of a psychological approach to care:

‘… We look in terms of your spiritual, your psychological intervention because we need to get their [*patients’*] buy-in before we can even start with the treatment. When you can’t get that, it’s very difficult to actually progress or even have a meaningful outcome at the end of your rehabilitation for this patient.’ (Participant 9, male, orthopaedic surgeon)

Social support was emphasised by the participants:

‘… to check the home circumstances as well as support … A patient who is going to need added support, where we have done the operation and they have got more than a pelvic fracture, and they cannot just non-weight-bear, or they are too weak to non-weight-bear, and then we had to put them in a wheelchair. They [*social workers*] will be the ones to be involved with the assessment of the home situation of the patient.’ (Participant 10, female, orthopaedic surgeon)

### Theme 6: Forging forward to improve care

The theme ‘forging forward to improve care’ includes interprofessional education (IPE) and collaborative practice (IPECP), student training, adopting the International Classification of Function (ICF), early management, patient education, standardisation of OMTs, patient-centred continued care and awareness campaigns, as sub-themes.

The majority of the participants highlighted a need for IPECP to improve the quality of healthcare:

‘As I have said, these patients have got associated injuries, so it is very important to collaborate with other specialities that can help us with the system that has been injured, for example the chest, the brain … haven’t had the experience of working with social workers and psychologists.’ (Participant 11, male, orthopaedic surgeon)

‘… no one [*patient*] will be missed. No patient will wait for the doctor to come … Every time you do a round, there are all those people [*HPs*] who are supposed to be there. They get to know the patients, they know the problems of the patient, they attend to them immediately.’ (Participant 15, female, professional nurse)

Participants also highlighted the importance of student training in improving HP collaboration:

‘I really think students should have patients together in an ideal world. The nutrition fourth year students and the physio fourth year students should maybe see on a weekly basis … So that they must start learning, so that when I am dietician and I am qualified and I work in an orthopaedic ward … I already have that background. So, I really think collaboration should start at student level.’ (Participant 7, female, dietitian)

Some participants suggested adopting the ICF for assessing and managing patients with pelvic fractures:

‘And if you look at the ICF model, it is the one that is actually going to assist us in trying to tackle this problem, but we need to get buy-in … It is there on paper but when you get into practice, there is still more of a medical model that is still in place in hospital settings; so that needs to change so that we are able to represent.’ (Participant 5, male, OT)

‘Because the physio does not ask questions regarding incontinence and regarding pain with sexual intercourse, regarding any of that, usually they do not pick it up that the patient has such problems.’ (Participant 2, female, physiotherapist)

The majority of participants identified early management as being important:

‘… as they [*patients*] come as an acute trauma, our protocol is that, if we need to do further investigations, like CT scan, we do those and we want to operate within the first 14 days of admission.’ (Participant 9, male, orthopaedic surgeon)

‘In an ideal world, I would like to start supplementation … a week or so before, ten days to seven days, before surgery, and then carry on afterwards. Because you really need the body a little bit stronger just before going into surgery.’ (Participant 7, female, dietitian)

Participants indicated the importance of patient-centred, continued care. Patient-centred, continued care puts emphasis on the individual patient’s needs, where the programme is tailored around the patient’s problems:

‘The other thing that I think will help a lot, is having progress on the patient. Because sometimes, some of the patients, once they are discharged, they tend to default. When they get home, they do not want to do bed rest or they want to get up and do other activities. So I think with follow-ups as well.’ (Participant 12, female, orthotist)

Most participants indicated a need for patient education:

‘I have realised, some of the patients, … default or they do not get well rehabilitated because they did not get proper education on their diagnosis. If we can get proper education in there, they will know and understand that.’ (Participant 12, female, orthotist)

‘… on-going orientation of patients about the hospital procedures and their rights and responsibilities to avoid problems and arguments between the patients and the treating staff.’ (Participant 13, male, social worker)

‘They [*patient*] don’t understand why some of the things have to be done on them and by them. So, most of them when they come to hospital, they feel that they are sick, they are not going to do anything by themselves … they [*patients*] have to know what to expect.’ (Participant 15, female, professional nurse)

The participants believed that using standardised OMTs for assessing the progress of patients would assist in healthcare:

‘… We have different questionnaires that deal with the different disorders. Like for an example, we have a comprehensive one that addresses incontinence – urinary incontinence, faecal incontinence, constipation, sexual dysfunction and, in women, prolapse. So, usually at first visit, we go through that questionnaire – they fill it in. With the follow up visits, we fill in another one, just to see if the symptoms have improved or not. And we also have quality-of-life questionnaires that they fill in on the first visit. And then on the last visit, when I feel that they are ready for discharge, I make them fill in another one – just to check if it has improved.’ (Participant 2, female, physiotherapist)

Other participants indicated the need for awareness campaigns:

‘I have mentioned that many of them [*patients*] are not seen. I just became aware of that during this interview. So, I will develop screening tools to identify patients with pelvic fractures, … I will strengthen the community work, just to make the communities aware of the pelvic fractures.’ (Participant 13, male, social worker)

‘… where you need talks … people from different departments can get together, and understand … even if it is one or two dieticians, one or two physios – get to know each other in a hospital. Not necessarily in a broad scale, but in a hospital. Bring the head of the pharmacy, the head of the kitchen, whoever – have them get together. And then have maybe a discussion. case studies, CPD activities, are always very helpful …’ (Participant 7, female, dietitian)

## Discussion

Healthcare professionals in this study when reflecting on the biopsychosocial lens of the patients indicated that patients with pelvic fractures usually present with impairments such as pelvic floor dysfunction and chronic pelvic pain, among others. Pelvic floor dysfunction included urinary incontinence and erectile dysfunction in men, whereas in women it included pelvic pain with sexual intercourse. Patients with pelvic fractures have a high risk of developing stress urinary incontinence as a result of the disruption of the pelvic floor.^16^ Sexual dysfunction is a common impairment in men, post-pelvic fractures^17^ and this may lead to functional limitations. In our study, sexual function is one of the activities of daily living (ADL) that is negatively influenced by pelvic fractures and this can consequently impact the patients’ relationship with their partners.^18,19^

Pelvic fractures lead to long-term impairments, including chronic pelvic and back pain, which affect the patient’s physical functioning, even several years following the injury.^20^ Chronic pelvic pain was one of the impairments noticed in our study and tends to affect gait and activities that involve sitting, standing, bending and running^16^ as well as activities around the home.^21^ Patients with pelvic fractures in SA report similar challenges. Discharged patients in a SA study reported difficulty in performing day-to-day activities, such as household chores, involvement in the community and earning an income, as a result of chronic pain and physical and emotional challenges.^4^ According to HPs, pain renders patients helpless and some end up resorting to the use of illegal substances for relief.

Healthcare professionals in our study felt that patients are discharged before they are functionally ready for community re-integration, and they are not yet independent enough, resulting in poor QoL. Furthermore, a lack of family support was identified as a barrier to rehabilitation and care. Patients with pelvic fractures often present with psychosocial complications^9^ making family support essential.

Care approach limitations emerged as impediments to service delivery. Healthcare professionals highlighted the poor use of comprehensive OMTs for evaluating, and as guidelines in the assessment and treatment of patients with pelvic fractures. These limitations were believed to compromise the quality of healthcare offered. Proper evaluations are essential for appropriate healthcare.^3^ The International Classification of Functioning (ICF), Disability and Health guides HPs in rendering effective healthcare.^22^ The literature indicates that the ICF guides the rehabilitation process, including setting goals, to provide HPs and patients with steps to follow.^23^ Studies have also used HRQoL-validated questionnaires for patients with pelvic fractures.^24^ These tools should be incorporated into the care approach to provide a holistic approach through a biopsychosocial lens.

Moreover, contextual impediments to care were highlighted. Healthcare professionals mentioned a shortage of staff, which makes it difficult to provide adequate healthcare for patients. The shortage of HPs is common in both developing and developed countries, and there is a limited number of skilled professionals in clinical settings, which compromises the quality of healthcare.^25^ A shortage of HPs leads to mental and physical exhaustion of staff because of the high number of patients managed within a short space of time.^26^ Furthermore, the unavailability of equipment and finances exacerbates an already pressurised staffing issue. The high number of patients puts further strain on the resources, including hospital beds,^27^ and leads to changes in the dynamics of hospital functioning, where patients might even be discharged earlier than anticipated.^28^ Interprofessional collaboration within the district and community is necessary for providing continuity of healthcare^13^ to curb complications associated with early discharge in patients.

Healthcare professionals reported on team challenges and highlighted a lack of understanding of different HP roles in the management of patients. To enhance team success, no profession’s contribution should be trivialised.^29^ Understanding the different HP roles promotes timeous referral of patients, thus leading to better collaborative care and, possibly, minimising secondary complications in patients. The referral system is about ensuring that patients reach the right HPs who are specialised in rendering appropriate and effective healthcare.^30^ Poor referral structures, mentioned in our study, lead to delayed interventions and treatment and possibly extended hospital stays.^3^

The lack of collaborative teamwork was highlighted in this study. Collaborative healthcare could assist with overcoming poor referrals and delayed patient management. Even though different HPs have unique roles, when working together, the team becomes more effective.^29,31^ Interprofessional collaboration should be strengthened to include the clinics and community health centres that offer services at the primary healthcare level,^13^ because patients need further support even after hospital discharge.

The biopsychosocial model of care is a framework that is encouraged during the management of patients with fractures in South Africa. This addresses the biological, psychological and social aspects of a patient’s life.^32^ In our study, HPs understood that physical rehabilitation to address impairments is part of a patient’s holistic healthcare needs.^3^ Using the necessary medical approach is crucial, as highlighted by the HPs in our research.^9^ However, social and psychological support was highlighted as a further necessity for patients. Psychosocial support included spiritual and psychological intervention prior to treatment, for optimal health outcomes to be achieved. Early intervention should include counselling patients to improve their full participation in therapy.^32^

Forging forward to improve care, HPs recommended the implementation of IPECP to improve care for patients with pelvic fractures. Patients with pelvic fractures often present with associated injuries, so a collaborative team approach will ensure holistic healthcare.^33^ Interprofessional education and collaborative practice fosters quality healthcare and improves patient outcomes.^31^ Collaboration should start in training, where students from different professions learn together through case studies. Bosch and Mansell^29^ advocate for the early introduction of IPE during student training because it promotes student teamwork and facilitates collaborative practice.

The HPs in our study advocated for standardised OMTs. The inclusion of patient-reported outcome measurements through the use of validated questionnaires throughout healthcare helps to stimulate conversations and identify patients who are at risk of complications.^19^ Patient-reported outcome measurements indicate how much a patient has benefited from the treatment^34^ and are preferable because they are based on the patient’s experiences about their status and thus reflect patient-centred care.^35^

The HPs in our study believed that the ICF would work well during assessing and managing patients with pelvic fractures. The ICF helps to standardise collaborative goal setting between HPs and patients^23^ and allows HPs to speak a common, health-related, understandable language.^22^ The ICF and IPECP include evidence-based practice and a biopsychosocial, socioecological and person-centred approach, and therefore complement each other in care approaches.^36^

Healthcare professionals emphasised the importance of early management of patients. Early, appropriate management and rehabilitation are important to curb the morbidity associated with the complexity of pelvic fractures.^9^ Moreover, early physiotherapy and rehabilitation reduce the number of possible complications in patients.^37^ Patient-centred care is necessary for individual patients to benefit maximally from the healthcare provided. Follow-up of discharged patients will ensure that they continue with their health programmes to promote a speedy recovery. Furthermore, patient-centred care empowers patients to play an active role in their management by adhering to their prescribed treatment, and ward and home programmes.^38^

Awareness campaigns were also recommended by HPs in the interviews to improve the care offered to patients with pelvic fractures. Various professionals could engage in discussions around pelvic fractures, as well as their roles and management approaches. Awareness campaigns could also improve knowledge and attitudes, and facilitate behaviour changes regarding health-related actions.^39^ Awareness campaigns should be extended as far as the district and the community level because both HPs and patients from the tertiary and district levels should be conversant about pelvic fractures and the challenges that are associated with them as highlighted in the study. Pelvic fractures are challenging to manage for HPs and, if not approached adequately, could burden the healthcare systems.^40^

## Conclusion

Our study revealed some challenges that influence healthcare quality for patients with pelvic fractures. However, strategies were also provided to improve the quality of healthcare. These strategies include the early management of patients; patient education; the inclusion of IPECP and undergraduate HP student training; adopting the ICF and the use of OMTs; as well as continued patient-centred care and awareness campaigns. Health education and promotion around pelvic fractures should be encouraged at the community, the district, and tertiary levels so that the community and HPs are all empowered. These recommended approaches could contribute to a holistic care approach to offer an integrated package of care to patients with pelvic fractures.

### Recommendations

This study highlighted the knowledge gap and recommends that more studies should be conducted on patients with pelvic fractures. Furthermore, the need for rehabilitation approaches and strategies to enhance the quality of life among patients with pelvic fractures is encouraged. The development of protocols and processes of interprofessional healthcare could further benefit services delivered to patients with pelvic fractures.

### Limitations of the study

Clinical psychologists and gynaecologists were not part of this study, despite several recruitment attempts. However, the researchers’ team had planned to include these HPs. It is believed that their views on the management of patients with pelvic fractures could have added great insight into the study. Poor network leads to postponing the interviews and providing an extra device with a different network provider on a different appointment.
